# Needs of Individuals Living With Hepatitis Delta Virus and Their Caregivers, 2016–2019

**DOI:** 10.5888/pcd17.200324

**Published:** 2020-12-17

**Authors:** Priyanka Kumar, Catherine Freeland, Sierra Bodor, Sean Farrell, Chari Cohen, Rosemary Frasso

**Affiliations:** 1 Sidney Kimmel Medical College at Thomas Jefferson University, Philadelphia, Pennsylvania; 2 Jefferson College of Population Health, Philadelphia, Pennsylvania; 3 Hepatitis B Foundation, Doylestown, Pennsylvania; 4 Geisinger Commonwealth School of Medicine, Scranton, Pennsylvania

## Abstract

**Introduction:**

Hepatitis delta virus (HDV) is a serious coinfection of the hepatitis B virus (HBV) that is estimated to affect between 48 to 72 million people worldwide. Data are limited on the informational needs of people living with HDV. The Hepatitis B Foundation, a US-based nonprofit organization that provides support to people living with HBV and HDV, receives emails (queries) as part of a helpline, a service to provide information, resources, and support to people affected by HBV and HDV.

**Methods:**

Query content was analyzed to assess the impact of HDV at the individual level. A total of 65 HDV-related queries from 17 countries were received from October 2016 to January 2019, and all were analyzed for this study.

**Results:**

Thematic analysis of queries indicated 4 dominant themes. Three were related to a need for information about 1) the disease and prevention of it, 2) disease symptoms and outcomes, and 3) treatment options. The fourth theme was related to barriers and quality of life. Individuals requested information on treatment options, medication access, diagnostic test interpretation, and clinical trials.

**Conclusion:**

Our study highlights the needs and lived experience of patients with HDV and summarizes critical information gaps. Findings can inform health care providers, public health professionals, and the pharmaceutical and biotechnology industries about the informational needs and lived experiences of individuals living with HDV and help create future HDV-related educational resources, care, and clinical trials.

**Summary**

**What is already known on this topic?**

Hepatitis delta virus (HDV) is a rare viral infection of the liver with potentially life-threatening consequences. Because of the rarity of the disease and a general lack of awareness, many patients may have gaps in critical disease-specific knowledge.

**What is added by this report?**

Little is known about the experiences of patients living with HDV. This qualitative study may be among the first to examine experiences of patients living with HDV and their caregivers to assess this population’s unique needs and challenges to care.

**What are the implications for public health practice?**

Identifying challenges to care and needs of HDV patients and their caregivers may help improve provider and public health practitioners’ ability to educate and care for this population.

## Introduction

Viral hepatitis accounts for an estimated 1.34 million deaths worldwide per year. Since 1990, viral hepatitis mortality has increased by 63%, and in 2017, hepatitis was the seventh leading cause of death in the world (1–4). Hepatitis delta virus (HDV) is a serious coinfection of HBV that is estimated to affect between 48 and 72 million people (13%–14.5%) (5–7) of the 292 million people living with chronic hepatitis B (CHB) worldwide (8).

Available data suggest that HBV–HDV coinfection is most prevalent in Central Asia, Eastern Europe, Central Latin America, and West and Central Africa (5–7). People most at risk for HBV–HDV coinfection are likely to be living in or have emigrated from these regions, have a history of intravenous drug use, are men who have sex with men, have HIV or hepatitis C virus (HCV), or have multiple sex partners (9,10). The rates of coinfection with HDV range from more than 10% to as high as 70% in countries in Africa, Asia, and parts of South America (11). In industrialized countries, such as Germany, England, and France, studies have shown recent increases in HDV prevalence (12,13). Epidemiologic and clinical research on HDV is sparse, contributing to an incomplete understanding of the actual disease burden, low global testing rates, and lack of effective treatments (12–14). Only people who already have CHB, or people who contract HBV and HDV through simultaneous exposure, can become infected (14), creating a defined risk group. Despite these factors, awareness among patients and providers is low, and treatment of HDV is far behind medical advancements for HBV and HCV (15,16). 

No US Food and Drug Administration–approved treatment of HDV exists, and the only somewhat effective treatment is pegylated interferon, with only 25% to 30% of patients able to control the virus with weekly injections administered for at least 1 year (17,18). When coinfection is poorly controlled, patients are 3 times as likely to develop cirrhosis and liver cancer, compared with HBV infection alone. Approximately 70% to 90% of coinfected patients develop cirrhosis within 5 to 10 years (19–22). Despite its discovery more than 40 years ago, knowledge of HDV is limited, and little is known about the informational needs and experiences of people living with HDV.

## Methods

We analyzed HDV-related email queries sent to the Hepatitis B Foundation, a US-based nonprofit organization, from October 2016 through January 2019 to identify information gaps and understand the lived experience of patients with HDV. The nonprofit organization receives queries as part of a free helpline, a service to provide information, resources, and support to people affected by HBV–HDV coinfection. The helpline is run by trained community health education specialists who are knowledgeable about HBV and HBV–HDV coinfection, and it provides patients with general guidance, disease information, and referrals to physicians or other health care providers.

All HDV-related queries received over 28 months were collected and de-identified by a trained researcher (S.P.). Queries that initially were not in English (n = 2) were translated using an online translation tool. Codes were developed in 2 ways: a priori (informed by the literature) and through line-by-line reading of a subsample of queries. Each code was given an explicit definition to ensure coding accuracy and improve intercoder reliability (23,24). Coding was performed by using NVivo12 software (QRS International), and all data were independently coded by 2 members of the research team (S.F., P.K.). Intercoder reliability was assessed by using the κ coefficient to identify coding discrepancies. The analysis team met (P.K., S.F., C.F., R.F.) throughout the coding process to discuss and resolve coding discrepancies until an acceptable final κ was achieved. Three of 19 codes had a mean κ of less than 0.75. These codes were examined in more detail by the analysis team so that coding discrepancies could be resolved, and κ scores were recalculated. This study was approved by the Heartland institutional review board and acknowledged by the Thomas Jefferson University institutional review board.

## Results

A total of 65 HDV-related queries were received from October 2016 to September 2019 and were included in this analysis. Most queries were from Romania (n = 11, 16.9%), Pakistan (n =10, 15.4%), and the United States (n = 9, 13.8%) (Table). Just over half of individuals identified themselves within the queries; of these, 31% self-identified as living with HBV–HDV coinfection (n = 20), and the remainder reported being caregivers, family, or friends of those with HDV (n = 10, 15%) or providers (n = 4, 6%). The mean adjusted final κ was 0.91 (range, 0.78–1.00), corresponding to near perfect intercoder agreement. Thematic analysis of queries identified 4 dominant themes, 3 related to a need for information about 1) the disease and its prevention, 2) disease symptoms and outcomes, and 3) treatment options.

**Table:** Frequency of HDV-Related Queries (N = 65) to the Hepatitis B Foundation^a^, by Country (n = 17), 2016–2019

**Country**	**Query Frequency (%) (n = 65)**
Romania	11 (16.9)
Pakistan	10 (15.4)
United States	9 (13.8)
Mongolia	4 (6.2)
Uzbekistan	3 (4.6)
Brazil	2 (3.1)
Ghana	2 (3.1)
Egypt	1 (1.5)
Finland	1 (1.5)
Germany	1 (1.5)
India	1 (1.5)
Kenya	1 (1.5)
Mauritania	1 (1.5)
Netherlands	1 (1.5)
Singapore	1 (1.5)
Spain	1 (1.5)
Uganda	1 (1.5)

Abbreviation: HDV, hepatitis delta virus.

a The Hepatitis B Foundation is a US-based nonprofit organization.

### Need for information on HDV diagnosis and prevention

People living with HDV and their family, friends, and caregivers often had questions about a new diagnosis of HDV, explicitly conveying questions about how to interpret diagnostic laboratory findings. For instance, one individual was unsure whether HDV-positive results meant a worsening or spread of an existing disease (HBV) or was a new infection. An example of this theme came from the following query: “I tested positive for HBV in 2012, and all I was told is that it would clear. No medication was prescribed. Yesterday I went for another test and was told that [it is] HDV. . . I do not understand what this means. [Is] this a new infection, or the disease is spreading?” After being diagnosed with both HBV and HDV, one patient wondered why their hepatitis results had drastically changed, despite being on medications, and asked for clarification of these results: “I have been on peg-Interferon for 12 weeks and Baraclude for 1 year. Now my enzymes are sky high, but my HBsAg has dropped to 26,000. At the same time, [my] HBV viral load has increased significantly. My question is, why has my viral load increased?”

Individuals receiving an initial diagnosis of HDV tended to have general questions about the disease. These questions asked either for advice on immediate next steps to take (eg, “What do I need to do?”) or for clarification about an HDV diagnosis (eg, “What [is] the meaning of delta virus?”) After receiving a diagnosis of HDV from their physician, another patient was notified of the rarity of the disease and re-emphasized the need for more information (eg, “Do you know of people with both hep B and D [and basal core mutation] or have any information on this?”) Patients and family members of those with HDV also had concerns about disease transmission. One query asked about how HDV was contracted in order to avoid the disease. Other patients and family members had questions about specific modes of disease transmission. For instance, another individual, the fiancé of someone living with HDV, had concerns about sexual transmission. This individual described, “My fiancé has just been presently diagnosed with hepatitis D infection. Am I safe or at risk of infection after sex/intimacy with him? Please, I need clarification.” Those living with HDV and family members wanted additional information about how to prevent HDV (“Please, how is hep D contracted and [what are] ways to avoid contracting the virus?”) or expressed confusion about an HBV–HDV diagnosis, despite taking preventive measures. “I kindly need [some] help to figure this [out,] as [I] am not sure why [I] was negative . . . got the preventive shots and all of a sudden now the results are showing positive as in having a chronic hep B infection.” A pregnant woman worried about transmission to her newborn and asked for methods to prevent potential harm from the disease for her child: “We’re expecting a baby in a month’s time. What can I do to protect the baby?”

### Understanding disease symptoms and outcomes

Patients with HDV described concerns about the risk of liver cancer or liver damage due to the disease. Sometimes patients asked about mechanisms through which HDV increased liver damage (eg, “I would like to know how hepatitis D [coinfection] comes about increasing the severity of the liver condition”) or shared information about newly diagnosed liver damage that surprised them. One participant stated, “I have what I’ve been told is a rare situation. Early this year tests came back that I had cirrhosis and another hepatitis, hep D (delta), along with the hep B, plus a basal core mutation, making me at high risk for liver cancer.” Often, patients and family members were curious about specific symptoms and wondered whether these could be attributed to HDV or other causes. “I have chronic hepatitis B and also ‘have’ hepatitis D (delta). I have multiple symptoms (abdominal pain, back pain, nausea, etc) that may or may not be related to my hepatitis. . . . We’ve been trying to determine the cause of my symptoms for years, thus if you happen to have any information on the above or are able to point me to where I can get more info, that would be greatly appreciated.”

### Treatment options

The need for clinical trials was the most salient aspect of “Treatment Options” and represented 16.9% (n = 11) of all queries that were included (N = 65). Patients and caregivers in many countries wanted to know about the availability of clinical trials in their own country and asked for information on how to register for and participate in these trials. One individual asked, “Is there [an] open phase 3 hepatitis delta clinical trial? Could you tell me how to register to participate?”

Patients also showed interest in finding curative treatments for HDV, especially when faced with a new diagnosis of the disease. “I have had hepatitis B for nearly 10 years and recently found out that I also have hepatitis D. I can’t say I am feeling well. Isn’t there any cure found for these?” Queries contained a variety of questions on medication, including recommendations for the newest and best medications, medication availability in certain countries, and advice on current medication regimens. The following is an excerpt from a query about the best medication recommendations after a patient was given a diagnosis of HDV.

“[Could] you please help me to find or recommend [to] me any latest best medication for this diagnosis?” Patients and family members of those with HDV were also very interested in treatment options. Questions included whether there were any treatments for HDV and what new treatments were available. “My mom is suffering from Hepatitis Delta. Is there any treatment for that virus?”

When faced with a new diagnosis of HDV, patients asked whether there were any lifestyle modifications they could make to improve the quality of life with the disease or discussed lifestyle modifications they had already implemented. These lifestyle modifications were often made in conjunction with medications and included eating healthy and abstaining from certain habits, such as smoking and drinking. “Right now, I take only those medicines, I try to eat healthy and I try to rest how much I can. I don’t drink alcohol and I don't smoke. Should I do other things?”

In addition to Western medicine or treatment modalities, some patients opted to try alternative therapies for their HBV–HDV, which included herbal or plant-based supplements. Patients using herbal or “natural” treatments often used these in conjunction with Western medications and reported using herbal treatments after they had tried other treatment options. “After I searched other treatment for my diseases, I discovered a clinic with natural drugs: SECOM. I was there and the doctor gave me a personal treatment. After 2 months taking these, ALT has normal value and AST is bigger with only 11 units.”

### Barriers to care and quality of life

Patients and caregivers or other family members sometimes noted different barriers to adequate care, including poverty, access to doctors or medication, or language barriers. Poverty was the most commonly noted reason for requesting help and more information. One individual described, “I need your help because [I] belong to a [low-income] family.” People living with HDV and their family members often felt worried, shocked, scared, and uncertain after receiving a diagnosis of HDV. One person living with HDV was very worried and expressed explicit concerns about how long they could live with the disease, stating, “I just found out that I have hepatitis B and D together. . . . How long can I live? What should I do? [Please], help me! I’m so scared.”

## Discussion

Qualitative analysis of queries received by the Hepatitis B Foundation indicated that people living with HDV and their family members had considerable concerns and the need for information that were related to 4 distinct thematic categories: 1) disease knowledge and prevention, 2) disease symptoms and outcomes, 3) treatment options, and 4) barriers and quality of life. People living with HDV and their family members and friends requested information on treatment options, medication access, diagnostic test interpretation, and availability of clinical trials. Questions about the availability of clinical trials and how to register for such trials was the most common topic among all queries.

To our knowledge, this study is the only one that qualitatively examines the experiences and needs of people living with HDV and their family members to assess needs and barriers in this population. Prior studies have used qualitative methods to explore various HBV patient experiences, including initial responses to disease, stigma associated with the disease, stress and anxiety about one’s health, and concerns about premature death (25–30), but to our knowledge none have examined the impact of HBV–HDV coinfection. This study showed similar sentiments and family members expressed worry about loved ones with HDV, which often manifested in searches for treatment options. People who received an initial diagnosis of HDV also expressed fear and anxiety, which was reflected in concerns about lifespan with the disease and confusion about the diagnosis. Despite these similarities, HDV patient experiences may be unique from those with HBV, HCV, or both because of the rareness of the condition and a lack of disease awareness among the general public. Future research should examine the differences between HDV patient experiences compared with HCV and HBV patient experiences.

Another notable commonality between this study’s findings and those of others is the lack of disease knowledge and lack of knowledge of treatment and screening options for viral hepatitis (27,29,31). People who sent queries about HDV had little knowledge of HDV but showed interest in furthering their knowledge, especially with regard to treatments and clinical trial research. These findings indicate the need for improved efforts to educate people who are diagnosed with HDV about their disease with the goal of increasing patient and caregiver knowledge. More knowledge about the disease among people living with HBV increases patient acceptance of disease and self-efficacy in disease management (29), which, based on our findings, could also be the case for those living with HDV. Future research should explore the relationship between HDV disease knowledge and patient self-efficacy in disease management.

Most qualitative literature about people living with viral hepatitis focuses on varied aspects of HBV and HCV patient experiences, in particular, the stigma associated with the disease, lack of disease knowledge, poor quality of life, and the complex emotions associated with having the disease. None of the queries we analyzed contained questions or content that referred to stigma or discrimination associated with HDV, possibly because patients had more immediate concerns about managing their health condition. No qualitative study has explored the needs and barriers of HDV patients, who may face more urgent needs related to treating and controlling disease progression (26,32), so more research is needed to understand stigma in the context of HDV.

In our study, people affected by HDV needed information about types of treatment options available to them, medication access, diagnostic test interpretation, and availability and location of clinical trials, the latter being the most common topic of all queries. These questions suggest that individuals with HDV may have challenges accessing medication, physicians with adequate knowledge about the disease, and clinical trials, and more work is needed to fully understand these challenges. Our findings also suggest that the subset of individuals who emailed queries specifically related to treatment may have already done online research about HDV before sending an email query. For example, many individuals who inquired about clinical trials mentioned hepatitis-specific medications and prepared detailed questions about interpretations of laboratory test results.

Results of this study emphasize the importance of targeted, patient-centered education for patients with HDV, and future education should focus on clarifying laboratory results, treatment options, medication access, and clinical trial resources for this population. Given the complex set of emotional, medical, and lifestyle factors that affect patients with HDV, materials should also be delivered accessibly and at the appropriate level of health literacy for those affected. Globally, patients with HBV–HDV coinfection may be more likely to be from more rural, isolated, or lower-income countries. Providers, especially those managing patients with HBV–HDV coinfection (primarily hepatologists), and public health professionals have a critical role in disseminating this information to patients because of their disease-specific knowledge and areas of expertise.

### Strengths and limitations

Our sample was people with HDV who sent email queries to a nonprofit helpline, so it may not be representative of the larger HDV-affected population. The queries analyzed in this study were limited to those received by the Hepatitis B Foundation. Many queries were from the United States, an industrialized country with a known lower incidence of HDV but with higher awareness. Because the Hepatitis B Foundation is US-based, Americans living with HDV and their caregivers may be more familiar with the foundation’s work and have more access to their resources, so our results are subject to regional bias.

Our data consisted only of email queries and did not include other forms of correspondence, such as in-person visits or queries received via mail, telephone, or social media platforms, so our sample may be less representative of the total population of patients with HDV. However, telephone calls to the Hepatitis B Foundation, are not recorded, so the ability to obtain these data was limited. Email queries were chosen for this analysis as the initial stage of this research. In the future, we hope to expand on this research to explore queries sent via social media platforms, including Instagram, Facebook, and Twitter. We expect that concerns will be similar to those of people using email. The sole use of email-based correspondence also may have affected sample representativeness because it limits the sample to people who have access to working internet (or electricity) and email accounts. Given the distribution of HDV in industrialized countries in Asia and Africa, many HDV patients may tend to live in less resource-rich environments. Our sample may not be representative of the larger HDV-affected population.

### Conclusion

This qualitative research indicated knowledge gaps among patients with HDV related to disease prevention, transmission risk to close family members, disease symptoms and long-term outcomes, and treatment options as well as barriers to care and overall quality of life. Our findings expand on the needs and barriers of individuals directly affected by HDV. A summary of these queries will be shared with providers who treat patients with HDV to help them expand their knowledge and understanding of the needs of their patients and their caregivers, and the data will be used to create more resources tailored to meet the unique needs of people living with HDV. Our findings may also be used to better understand the information needs, challenges, and quality of life implications of patients with HDV to inform clinical trial designs and the development of new treatments. Like other chronic viral hepatitis patients, those affected by HDV need more education on certain aspects of their disease. Although our findings are not representative of the larger HDV global population, we believe that they support the claim of a larger knowledge gap that exists surrounding HDV. Because people living with HDV and their family members tended to need more information on treatment options, medication access, diagnostic test interpretation, and clinical trials, providers and public health organizations therefore have the most crucial role in educating people with HDV and their families. Ultimately, targeted education interventions for this patient population are important because they can improve self-efficacy in disease management and overall quality of life. Future research is needed to fully understand the impact HDV has on the lives of people who are affected, and the knowledge gained from this research will help guide future research projects and outreach initiatives for those affected by HDV. We hope our findings can help educate providers, public health professionals, and the pharmaceutical and biotechnology industries on the needs of individuals living with HDV, so we can collectively address them.
